# Non-traumatic splenic rupture in a patient on oral anticoagulation

**DOI:** 10.1186/1865-1380-6-16

**Published:** 2013-05-21

**Authors:** Marije M de Kubber, Lucia JM Kroft, Bas de Groot

**Affiliations:** 1 Department of Emergency Medicine, Leiden University Medical Centre, Albinusdreef 2, 2333 Leiden, ZA, the Netherlands; 2 2Department of Radiology, Leiden University Medical Centre, Albinusdreef 2, 2333 Leiden, ZA, the Netherlands

**Keywords:** Non-traumatic splenic rupture, Spleen, Oral anticoagulation, Epigastric pain

## Abstract

**Background:**

Splenic injury is normally associated with trauma, but spontaneous splenic rupture has been described in various systemic diseases.

**Case presentation:**

A 56-year-old male on oral anticoagulation presented to the emergency department with epigastric pain, nausea, and left upper quadrant tenderness. There was no history of trauma. Contrast-enhanced CT imaging revealed a large subcapsular haematoma of the spleen. Oral anticoagulation was antagonised with vitamin K and the patient was discharged in good condition after 3 days of clinical observation.

**Conclusion:**

Non-traumatic splenic rupture is a rare complication of oral anticoagulation.

## Findings

Spontaneous splenic rupture is a rare and life-threatening condition. It is associated with pre-existent splenic pathology and various disease entities. Few reports have described the association with oral anticoagulation. Treatment normally consists of discontinuation of oral anticoagulation and surgery for patients in shock [1,2].

## Case presentation

**Figure 1:**
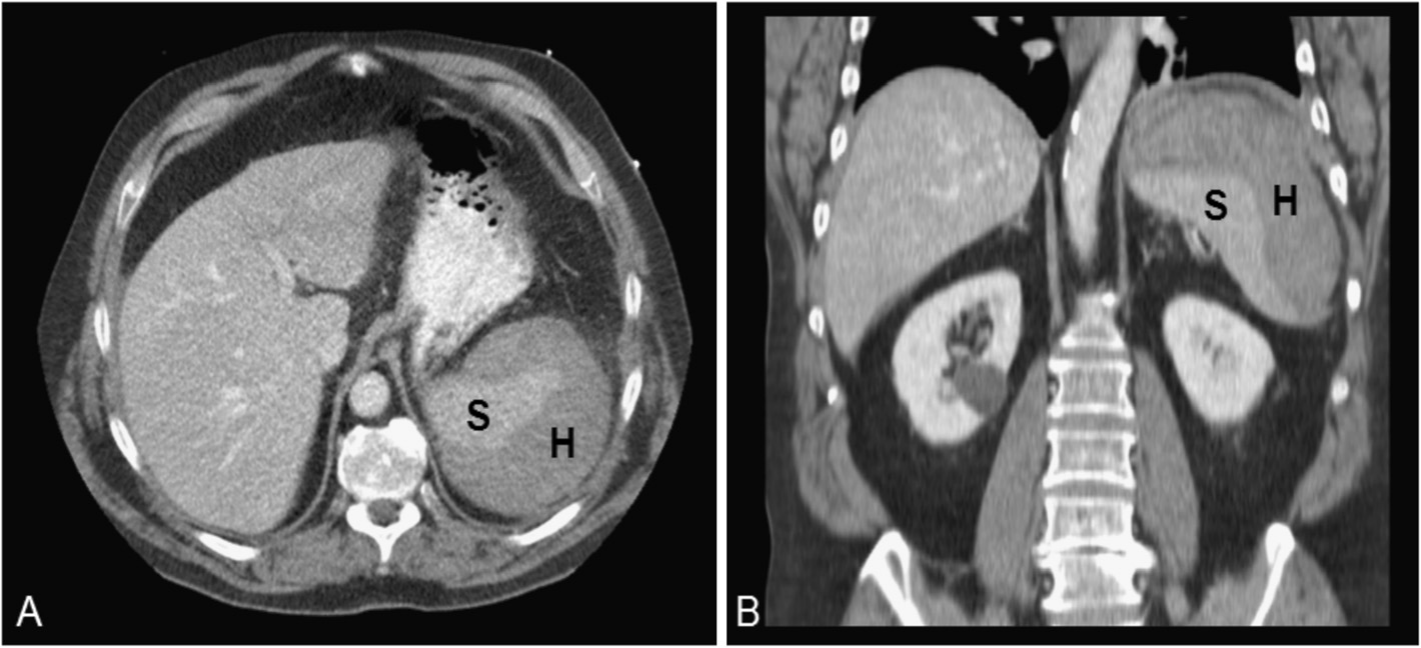
**Contrast-enhanced computed tomography image with 5-mm slides in transverse (A) and coronal (B) orientation.** Spleen (S) with large subcapsular hematoma (H) with an estimated volume of 0.75 l.

A 56-year-old Caucasian male with a past medical history of hypertension, acute coronary syndrome and atrial fibrillation presented to the emergency department (ED) with acute epigastric pain. His complaints had started 2 weeks earlier and had worsened 1 day prior to ED presentation. The pain was associated with nausea and increased on inspiration. Defecation and micturition were normal. In addition to fenprocoumon and sotalol, he was on antihypertensives, a statin and a proton-pump inhibitor. There was no history of trauma. Physical examination showed a pale, sweating and obese man in pain. His blood pressure was 113/73 mmHg, with a heart rate of 72 beats/min, oxygen saturation of 95% on room air and a respiratory rate of 13 breaths/min. The abdomen was not distended, and there were normal bowel sounds. He had epigastric tenderness without muscular defence or hepato-splenomegaly. His initial haemoglobin was 8.5 mmol/l. White blood cell count was 8.5 × 10^9^/l with a c-reactive protein of 26 mg/l. The international normalised ratio (INR) was 2.4. Abdominal ultrasound showed an inhomogeneous aspect of the spleen without free fluid. Contrast-enhanced computed tomography (CT) imaging of the abdomen revealed splenic haemorrhage with subcapsular hematoma (Figure 1).

An acute operation was deemed unnecessary because his vital signs remained stable. Oral anticoagulation was reversed with 10 mg vitamin K. The patient was admitted to the hospital for observation and was discharged in good condition after 3 days.

## Discussion

Non-traumatic splenic rupture may occur as a complication of neoplastic, infectious, inflammatory and genetic disorders. Various drugs and treatment modalities have also been associated with non-traumatic splenic rupture. In the presented case no other cause except for oral anticoagulation was found.

Mortality is relatively low when an underlying aetiology is absent but can be as high as 12.2% when caused by an underlying disease [3]. Patients typically present with abdominal pain, referring pain to the left shoulder and sometimes shock [1].

Radiologic investigations play a major role in diagnosing spontaneous splenic injury. CT imaging has a sensitivity and specificity of at least 95% [4].

Haemodynamically stable patients with non-traumatic splenic rupture are normally treated conservatively, while patients with active bleeding or shock usually require an operation, according to the guidelines of the Eastern Association of Surgery of Trauma (EAST) [2].

## Conclusion

Non-traumatic splenic rupture is a rare complication of oral anticoagulation.

## Competing interests

The authors declare that they have no competing interests.

## Authors’ contributions

MM carried out the literature research and wrote the manuscript. LJM provided the imaging picture. LJM and B provided critical revision of the manuscript. All authors read and approved the final manuscript.
